# Novel Non-surgical Strategy of Severe Chest Trauma With Venovenous Extracorporeal Membrane Oxygenation, Angioembolization, and Bronchial Blocker: A Case Report

**DOI:** 10.7759/cureus.58359

**Published:** 2024-04-16

**Authors:** Tomohiro Morito, Yosuke Matsumura

**Affiliations:** 1 Department of Intensive Care, Chiba Emergency and Psychiatric Medical Center, Chiba, JPN

**Keywords:** extracorporeal membrane oxygenation, bronchial blocker, lung contusion, interventional radiology, chest trauma

## Abstract

Severe chest trauma often requires immediate intervention, typically involving open chest surgery. However, advancements in medical technology offer alternative approaches, such as endovascular therapy and venovenous extracorporeal membrane oxygenation (VV-ECMO). In a recent case, a middle-aged male cyclist was admitted after colliding with a vehicle, presenting in shock with multiple injuries, including cerebral contusion and rib fractures. Despite initial treatments such as chest tubes and blood transfusions, his condition remained unstable, with worsening respiratory failure and hemorrhagic shock.

A multidisciplinary team devised a comprehensive treatment plan, utilizing VV-ECMO for oxygenation support, a bronchial blocker to localize the hematoma, and interventional radiology for hemothorax hemostasis. These interventions successfully stabilized the patient without resorting to open chest surgery. Endovascular therapy, alongside bronchial blockers, facilitated adequate hemostasis and hematoma localization, avoiding invasive procedures. VV-ECMO plays a crucial role in maintaining oxygenation during respiratory failure. Strategic anticoagulation with nafamostat mesylate prevented clotting in the ECMO circuit.

This case highlights the effectiveness of minimally invasive strategies in managing severe chest trauma, preserving lung function, and improving outcomes. In refractory cases, VV-ECMO acts as a bridge to stabilize respiratory status before definitive treatment, while bronchial blockers localize hematomas, reducing the need for surgery. Interventional radiology offers a less invasive option for achieving hemostasis. Collaboration among medical specialties and innovative technologies is critical to successfully navigating complex chest trauma cases.

## Introduction

Most severe chest trauma cases do not need any open surgical procedures; only 30% of penetrating chest injuries and 10% of blunt chest injuries require hemostatic surgery [1,2]. The standard hemostatic strategy for severe chest trauma has been open chest hemostasis. On the other hand, the importance of endovascular therapy, such as hemostasis by interventional radiology (IR) [3] and respiratory support by venovenous extracorporeal membrane oxygenation (VV-ECMO) for respiratory failure, has been recognized [4].

VV-ECMO could be considered a rescue treatment for the management of severe hypoxemic respiratory failure [5]. A recent report demonstrated that early VV-ECMO did not have increased mortality [4]. Massive hemoptysis can lead to death by suffocation. A bronchial blocker is usually used for one-lung ventilation [6,7] but can also be used for bronchial occlusion in blunt chest trauma [8,9]. However, the use of bronchial blockers has a risk of hypoxemia, malposition, and iatrogenic bronchial injury.

In this report, we describe a successful case of severe chest trauma presenting respiratory failure and hemorrhagic shock, using emergent VV-ECMO to support the oxygenation, a bronchial blocker to localize the hematoma, and IR to achieve the hemostasis of the hemothorax. We also clarify the circumstances under which these interventions are most beneficial and elaborate on their roles in mitigating complications.

## Case presentation

A male bicycle rider in his 40s collided with a motor vehicle. At the time of emergency medical service contact, he presented shock with unmeasurable blood pressure and impaired consciousness and was transferred to our hospital. He had a history of cerebral contusion, skull fracture, hydrocephalus after ventricular-peritoneal shunt implantation, symptomatic epilepsy, old multiple rib fractures, and bronchial asthma.

The primary survey showed that the airway was barely open, but it revealed moderate tachypnea at 23 breaths/minute and unmeasurable SpO_2_ under a 10 L reservoir mask with bilateral breath sounds. The radial artery was not palpable, heart rate was 119 beats/minute, systolic blood pressure was 80 mmHg, and extended focused assessment with sonography for trauma (EFAST) [10] was negative for abdomen and pericardiac effusion, and poor images for chest assessment. Glasgow coma scale score was 3 (E1V1M1), pupil diameter was 3 mm/3 mm (no light reflex), and body temperature was 35.3°C.

**Figure 1:**
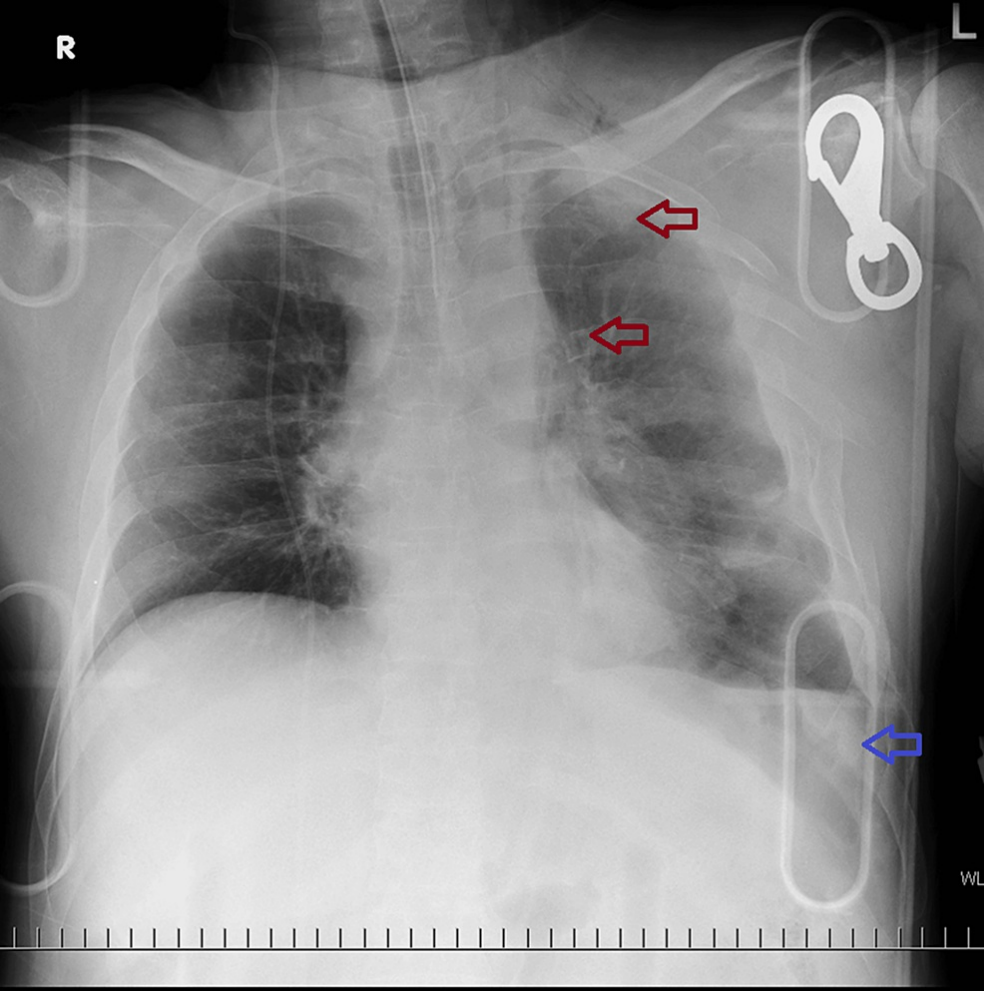
Chest X-ray on arrival. Bilateral multiple rib fractures (red arrow) and hemopneumothorax (blue arrow).

Due to respiratory failure, shock, and impaired consciousness, we intubated the patient to secure the airway and provide positive pressure ventilation. A chest X-ray revealed bilateral multiple rib fractures and left hemopneumothorax (Figure 1). A left thoracic drain was placed, and 500 mL of blood was drained.

**Figure 2:**
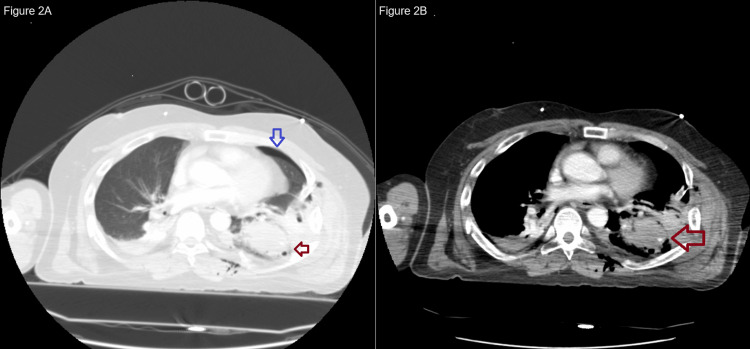
CT of the lung. Left lung contusion (red arrow) and pneumothorax (blue arrow). Extravasation is seen in the contusion.

**Figure 3:**
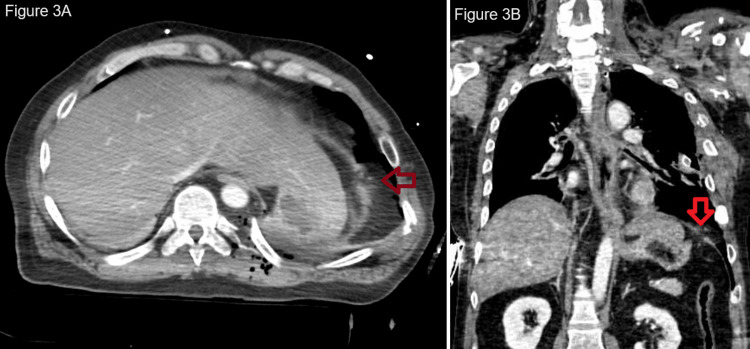
CT image presenting left traumatic diaphragmatic injury (red arrow).

With the response to the initial fluid and the massive transfusion order, he was moved to the CT scan. A trauma pan-scan showed bilateral multiple rib fractures with flail chest (right 1-7, left 1-11), left hemopneumothorax, bilateral lung contusions (Figure 2), and left diaphragmatic injury (Figure 3, Video 1). The left lung contusion contained contrast extravasation (Figure 2). The remaining injuries included the following: a right temporal artificial bone fracture, a left mandibular process fracture, a cervical transverse process fracture (left 5th-7th), bilateral distal clavicle fractures, a right scapula body fracture, a right proximal humerus fracture, and a left humeral avulsion fracture. The Injury Severity Score was 24 (chest Abbreviated Injury Scale (AIS), 4; cervical spine AIS, 2; upper extremities AIS, 2), the Revised Trauma Score was 1.90, and the probability of survival was 0.28.

**Video 1:** CT images of the chest and abdomen.

**Figure 4:**
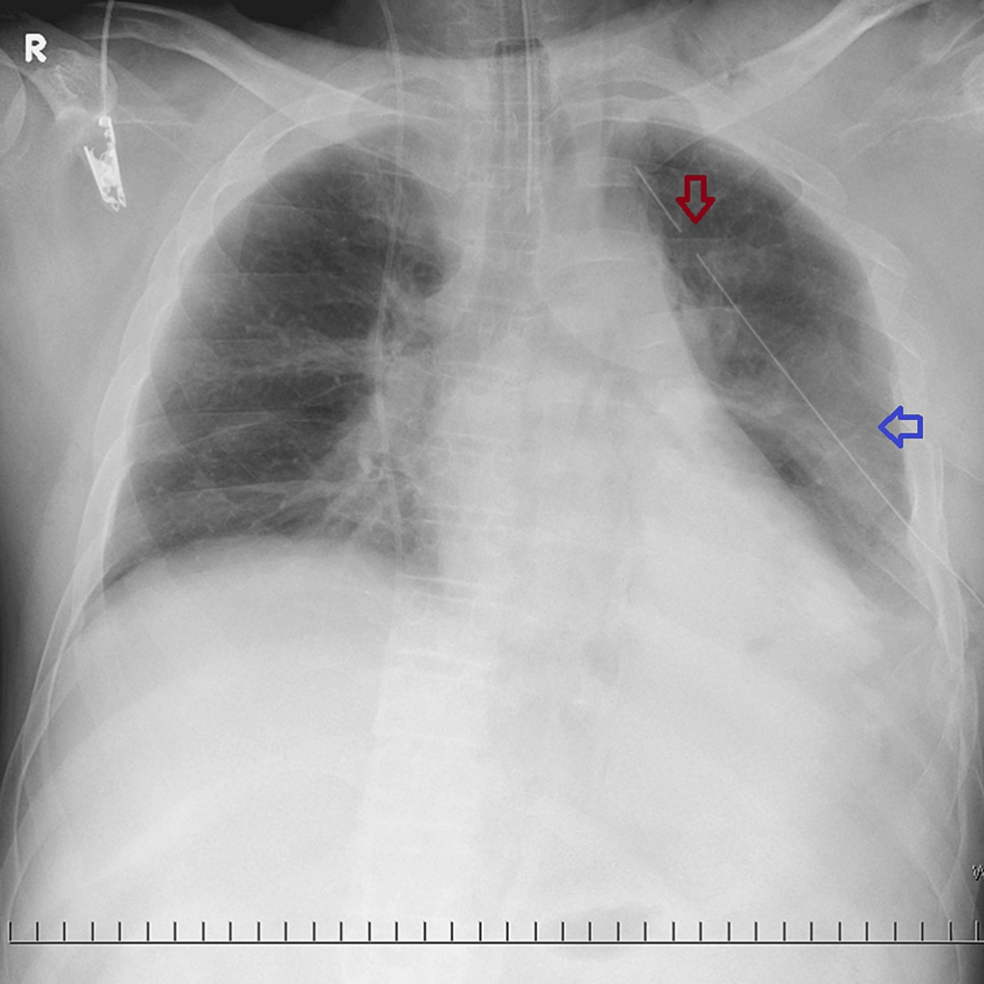
Chest X-ray before extracorporeal membrane oxygenation introduction. The left hemothorax was drained with a chest tube (red arrow). Left lung contusion deteriorated (blue arrow).

The blood type of the patient was revealed as O-negative, which is extremely rare in Japan. Thus, we hesitated to initiate massive transfusion using O-positive red blood cells, resulting in the delay of transfusion and unavoidable vasopressor use. Despite blood transfusions and vasopressor, the patient remained hemodynamically unstable and continued to drain blood at a rate of 200 mL/hour from the chest tube. A small amount of bloody sputum was suctioned from the endotracheal tube. The PaO_2_/FIO_2_ ratio dropped to 110 (pH = 7.214, pCO_2_ = 42.0 mmHg, pO_2_ = 110 mmHg, HCO_3_ = 16.3 mmol/L) (ventilator settings: volume control ventilation: FIO_2_ = 1.0, tidal volume (TV) = 600 mL, positive end-expiratory pressure (PEEP) = 10 cmH_2_O, frequency: 10 cycles/minute). We determined that the patient had progressive respiratory failure and prolonged hemorrhagic shock due to hemothorax and pulmonary contusion. Repeat EFAST and chest X-ray did not show tension pneumothorax. Respiratory failure due to pulmonary contusion was more lethal than unstable hemodynamics due to massive ongoing hemothorax. We recognized the deterioration of lung laceration associated with hematocele in the lung parenchyma based on the repeat chest X-ray (Figure 4) and continuous hemorrhage from the endotracheal tube.

With the recommendation of a trauma surgeon and simultaneous preparation of surgery, we planned to maintain oxygenation with VV-ECMO, localize the hematoma with a bronchial blocker in the left main bronchus to avoid massive hemoptysis, and embolize the intercostal and bronchial arteries with IR.

VV-ECMO was initiated without anticoagulation configured with a 25 Fr drainage cannulae in the right internal jugular vein and a 20 Fr returning cannulae in the right femoral vein in the angiography suite.

**Figure 5:**
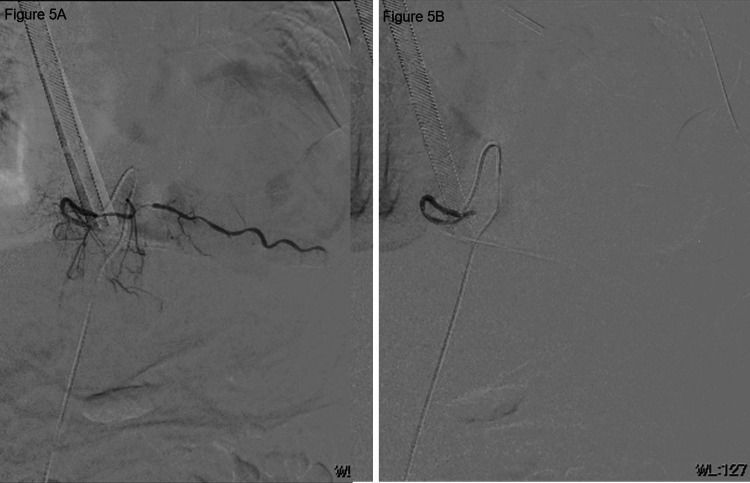
Angiography image of the left sixth intercostal artery. A: pre-embolization; B, post-embolization.

**Figure 6:**
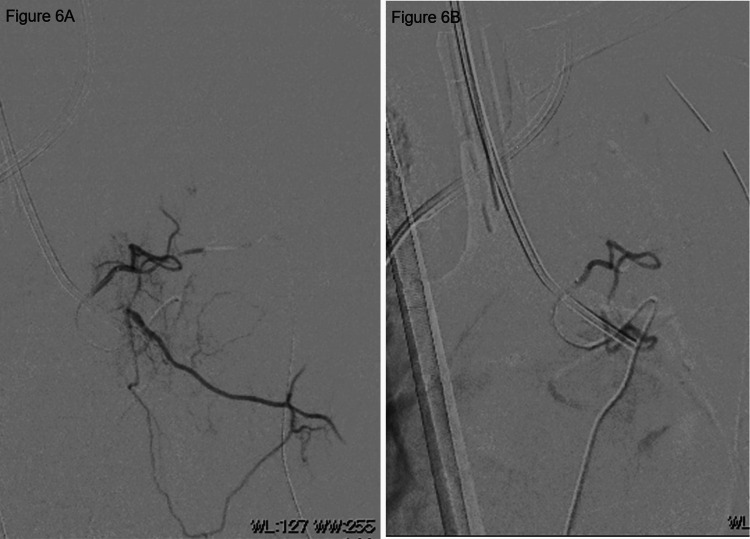
Angiography image of the left third intercostal artery and left bronchial artery. A, pre-embolization; B, post-embolization.

The ventilator settings were changed to the following: pressure control ventilation: FIO_2_ = 0.5; PEEP = 10 cmH_2_O; peak inspiratory pressure = 25 cmH_2_O, frequency = 10 times/minute, inspiratory time = 1.2 seconds. A 9 Fr bronchial blocker (Phycon TCB bronchial blocker, Fuji Systems Corporation, Tokyo, Japan) was advanced through a multi-port airway adapter connected to the endotracheal tube into the left main bronchus with bronchoscopic guidance. The balloon was inflated to localize the hematoma by the tamponade effect. The maximum inflation volume was 8 mL, determined with bronchoscopic confirmation. Angiography was subsequently performed from the right femoral artery. The left superior intercostal artery to the sixth intercostal artery was embolized with gelatin sponges (Figures 5, 6). After these endovascular procedures, the patient was admitted to the intensive care unit.

**Figure 7:**
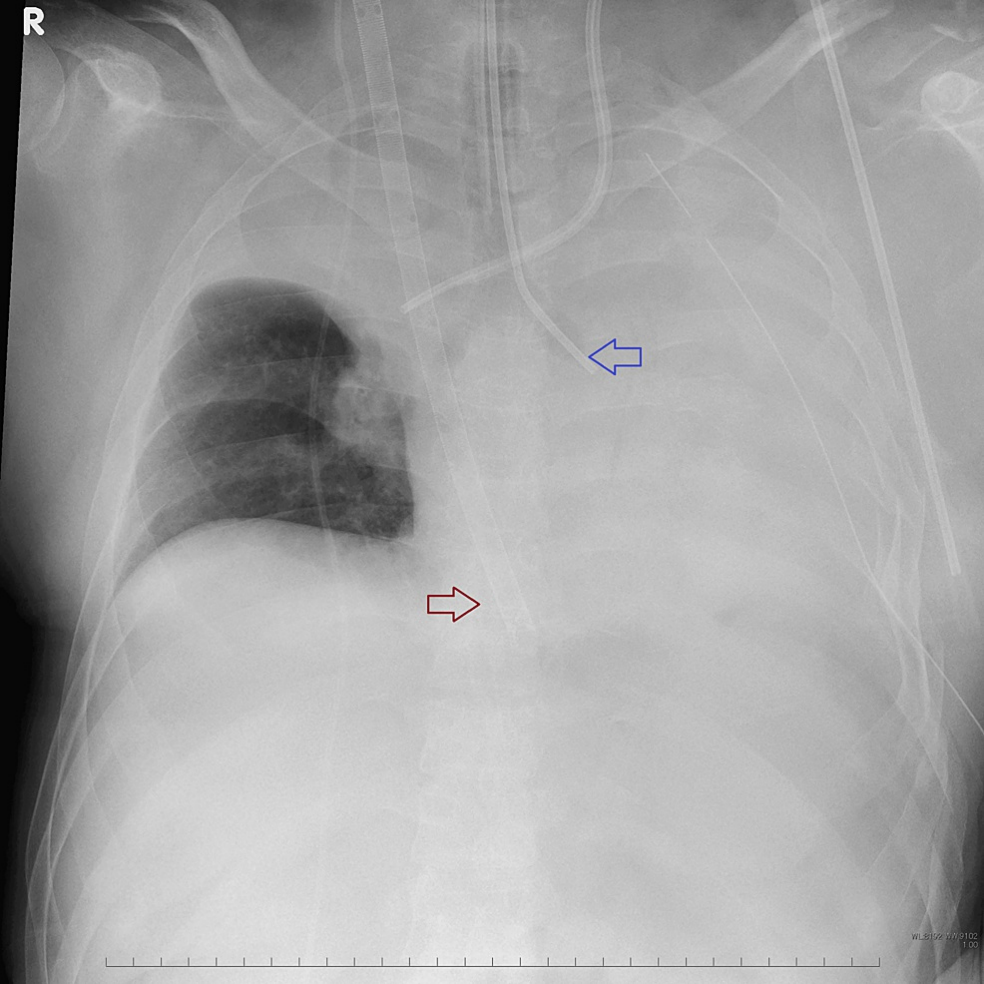
Chest X-ray after venovenous extracorporeal membrane oxygenation (red arrow) and a blocker placement (blue arrow). The chest X-ray demonstrated total atelectasis of the left lung with a blocker.

He received transfusions to correct the coagulopathy, and 16 units of packed red blood cells and 18 units of fresh frozen plasma were administered within 24 hours of arrival. The chest X-ray demonstrated total atelectasis of the left lung with a blocker (Figure 7), suggesting the tamponade hemostasis of the left lung contusion with VV-ECMO support. By the second hospital day, correction of the coagulopathy was complete, and bloody drainage from the thoracic drain had decreased.

As thrombus began to form in the ECMO circuit, anticoagulation was started with nafamostat mesylate (NM) and adjusted to an activated partial thromboplastin time of 40 to 50 seconds. The bronchial blocker was deflated under bronchoscopic assistance, and no apparent eruptive bleeding was observed. The bronchial blocker was withdrawn, and sputum aspiration and removal of blood clots were repeated using a bronchoscope. No rebleeding of either hemothorax or pulmonary contusion was observed after NM started.

His respiratory status improved, and he was weaned from VV-ECMO on the third day after an ECMO clamp test confirmed that oxygenation could be maintained with ventilation from his own lungs alone. He had severely displaced rib fractures resulting in the sustained flail chest. During ventilatory management, he underwent bilateral clavicle and left rib fixation on the fifth day. On the sixth day, laparoscopic diaphragm repair was performed to prevent diaphragmatic herniation during spontaneous breathing. On the 13th day, he underwent an open repair of the right humeral neck fracture and left rib remobilization. The patient was extubated on the 15th day after confirming that there was no flailing of the thorax during spontaneous respiration. On the 18th day, the patient was transferred to the general ward, and on the 51st day, he was transferred to the hospital for rehabilitation on his own.

## Discussion

A patient with severe chest trauma presenting with respiratory failure and hemorrhagic shock was saved by a combination of endovascular therapy and bronchial blocker instead of open chest surgery. Oxygenation was maintained with VV-ECMO and tamponade hemostasis, and hematoma localization was achieved with bronchial blockers. The intercostal and bronchial arteries were embolized to stop bleeding. A patient with pulmonary contusion with extravascular leakage and hemothorax due to multiple rib fractures was successfully treated without pneumonectomy.

The standard treatment for massive hemothorax is thoracotomy, which is mandatory when the source of bleeding is the lung parenchyma or great vessels. A hemodynamically unstable patient with continuous active chest drainage should be the immediate surgical candidate in standard trauma care. Nevertheless, we determined that his oxygenation was too critical to undergo thoracotomy and lung resection. We recognized that severely displaced multiple rib fractures are the primary source of hemothorax rather than lung contusion because extravasation in the lung contusion was localized in the lung parenchyma. When the bleeding source of hemothorax is from the chest wall, adequate hemostasis can be expected with IR instead of thoracotomy, and its usefulness has been reported [3,11]. Based on the diagnosis of hemothorax due to multiple rib fractures on CT angiography, we chose IR as the best means of hemostasis in this case because angioembolization is often more effective and faster than surgical ligation of the ribs.

When extravasation is present in a pulmonary contusion, airway hemorrhage can expand, resulting in respiratory failure as well as hemorrhagic shock. Non-anatomic lung resection of a contused area is the standard treatment option, but lung resection during severe respiratory failure leads to further deterioration of respiratory function. In the present case, we introduced VV-ECMO urgently because respiratory failure progressed, and oxygenation could no longer be maintained with ventilatory management.

VV-ECMO is a treatment option in patients with severe hypoxemic respiratory failure [5], which requires endovascular intervention, and usually anticoagulant is administered. The most serious concern in using VV-ECMO in trauma patients is a potential increase in hemorrhage. The shorter time from injury to cannulation for VV-ECMO was associated with death in a previous study [12]. However, initially, heparin-free ECMO [13] and a dedicated multidisciplinary intensive care unit [14] may overcome the management difficulty. Early VV-ECMO did not increase mortality compared with the overall trauma VV-ECMO population [4]. VV-ECMO was also reported as a treatment strategy for respiratory stabilization in chest trauma with respiratory failure, with pulmonary contusions accounting for the most significant number of cases [4]. Unlike hemothorax and pneumothorax, pulmonary contusions are not amenable to thoracic drainage. Although tracheal intubation and positive pressure ventilation effectively expand the lungs, respiratory failure progresses as bleeding from the contusion flows into the healthy lung parenchyma. Thus, severe pulmonary contusions can progress to respiratory failure requiring VV-ECMO. To manage VV-ECMO in the setting of hemorrhagic shock, abundant blood flow must be ensured; placement of a large 25 Fr or larger drainage cannulae in the right internal jugular vein ensures a stable high flow rate of 4-5 L/minute. The high blood flow rate decreases thrombus formation in the circuit without anticoagulation. After control of bleeding and coagulopathy, NM, which has a shorter half-life than heparin, can be used for management with minimal risk of bleeding complications [15].

Preventing airway hemorrhage spread is essential to treat pulmonary contusions without lung resection. Bronchial blockers localize the hematoma [6]. The effects of bronchial blockers are tamponade hemostasis and the protection of the healthy lung [9]. In theory, blocker placement in the left lower lobe branch is more selective in blocking. However, it becomes ineffective if the bronchial blocker is moved to the left main bronchus due to patient transfer or repositioning. We placed the blocker in the left main bronchus for a stable block. Compared to a double-lumen tube, which is often difficult to intubate, the bronchial blocker placement has advantages in terms of speed and safety because it can be used through a regular endotracheal tube. In a previous report, placing a bronchial blocker in a pulmonary contusion with active bleeding minimized the hematoma extension to other areas in the lung, prevented deterioration of respiratory status, and saved the patient’s life [8].

Combining endovascular therapy such as VV-ECMO and IR with bronchial blockers, a patient with pulmonary contusion and hemothorax who presented with respiratory failure and hemorrhagic shock was saved without open chest surgery. The fluoroscopic environment allows for the treatment of patients with severe chest trauma with preservation of pulmonary function. By accumulating experience in endovascular treatment in addition to standard surgical care, the development of robust management protocols applicable to severe chest trauma with critical oxygenation is expected in the future.

## Conclusions

In severe chest trauma refractory to ventilatory management, oxygenation can be maintained with VV-ECMO to stabilize respiratory status before definitive hemostasis. ECMO management should be initiated without anticoagulation with large debridement vessels to ensure abundant blood flow, and NM should be selected after bleeding control. Bronchial blockers for pulmonary contusion localize the hematoma and help avoid pneumonectomy. IR can effectively stop hemothorax of chest wall origin. Endovascular therapies such as ECMO and IR are minimally invasive and lung function-sparing strategies in severe chest trauma.

## References

[1] (2018). ATLS®: Advanced Trauma Life Support Student Course Manual. American College of Surgeons.

[2] Feliciano DV, Mattox KL, Moore EE (2020). Trauma, 9e. NY.

[3] Tamburini N, Carriel N, Cavallesco G (2019). Technical results, clinical efficacy and predictors of outcome of intercostal arteries embolization for hemothorax: a two-institutions' experience. J Thorac Dis.

[4] Powell EK, Reynolds TS, Webb JK (2023). Early veno-venous extracorporeal membrane oxygenation is an effective strategy for traumatically injured patients presenting with refractory respiratory failure. J Trauma Acute Care Surg.

[5] Robba C, Ortu A, Bilotta F, Lombardo A, Sekhon MS, Gallo F, Matta BF (2017). Extracorporeal membrane oxygenation for adult respiratory distress syndrome in trauma patients: a case series and systematic literature review. J Trauma Acute Care Surg.

[6] Campos JH (2003). An update on bronchial blockers during lung separation techniques in adults. Anesth Analg.

[7] Palaczynski P, Misiolek H, Szarpak L (2023). Systematic review and meta-analysis of efficiency and safety of double-lumen tube and bronchial blocker for one-lung ventilation. J Clin Med.

[8] Memtsoudis SG, Sadovnikoff N (2007). Successful management of a trauma patient with pulmonary hemorrhage using a wire-guided bronchial blocker. J Trauma.

[9] Nishiumi N, Nakagawa T, Masuda R, Iwasaki M, Inokuchi S, Inoue H (2008). Endobronchial bleeding associated with blunt chest trauma treated by bronchial occlusion with a Univent. Ann Thorac Surg.

[10] Desai N, Harris T (2018). Extended focused assessment with sonography in trauma. BJA Educ.

[11] Hagiwara A, Yanagawa Y, Kaneko N, Takasu A, Hatanaka K, Sakamoto T, Okada Y (2008). Indications for transcatheter arterial embolization in persistent hemothorax caused by blunt trauma. J Trauma.

[12] Menaker J, Tesoriero RB, Tabatabai A (2018). Veno-venous extracorporeal membrane oxygenation (VV ECMO) for acute respiratory failure following injury: outcomes in a high-volume adult trauma center with a dedicated unit for VV ECMO. World J Surg.

[13] Arlt M, Philipp A, Voelkel S (2010). Extracorporeal membrane oxygenation in severe trauma patients with bleeding shock. Resuscitation.

[14] Menaker J, Dolly K, Rector R (2017). The lung rescue unit-does a dedicated intensive care unit for venovenous extracorporeal membrane oxygenation improve survival to discharge?. J Trauma Acute Care Surg.

[15] Lang Y, Zheng Y, Qi B (2022). Anticoagulation with nafamostat mesilate during extracorporeal life support. Int J Cardiol.

